# Healthcare-associated infections in Africa: a systematic review and meta-analysis of point prevalence studies

**DOI:** 10.1186/s40545-022-00500-5

**Published:** 2022-12-09

**Authors:** Usman Abubakar, Omalhassan Amir, Jesús Rodríguez-Baño

**Affiliations:** 1 Discipline of Clinical Pharmacy, School of Pharmaceutical Sciences, Universiti Sains Malaysia, 11800 Penang, Malaysia; 2 Department of Clinical Pharmacy, International University of Africa, Khartoum, Sudan; 3 Infectious Diseases and Microbiology Division, Department of Medicine, Hospital Universitario Virgen Macarena, Biomedicine Institute of Seville (IBiS)/CSIC, University of Seville, Seville, Spain; 4 CIBERINFEC, Instituto de Salud Carlos III, Madrid, Spain

**Keywords:** Health-associated infections, Hospital-acquired infections, Point-prevalence, Africa, Infection prevention and control, Systematic review, Meta-analysis

## Abstract

**Background:**

There is limited data to describe the point-prevalence of healthcare-associated infections (HAIs) among patients at a regional level in Africa. This study estimated the pooled prevalence of HAIs and described the distribution of HAIs as well as the pathogens identified from African studies.

**Methods:**

PubMed, Scopus and Google Scholar databases were searched to find point-prevalence studies of HAIs in Africa. Studies conducted in Humans that reported the prevalence of HAIs among hospitalized patients and published in English language from January 2010 to March 2022 were selected. Longitudinal studies of HAIs and unpublished studies were excluded. The reference list of the selected studies was checked to find additional studies. A meta-analysis was conducted using RevMan 5.4 and the pooled prevalence of HAIs was determined using a random effect model.

**Results:**

Of the 6094 articles identified from the databases, fifteen eligible articles were selected. The studies were conducted in the North, South, East and West African regions with Tunisia (*n* = 4) and South Africa (*n* = 2) having the highest number of studies. Most of the studies (*n* = 12, 80.0%) had good quality. The pooled prevalence of HAIs was 12.76% (95% confidence interval [CI] 10.30–15.23) with a high degree of heterogeneity (*I*^2^ = 90.0%). The prevalence of HAIs varied between wards with the highest rate found in the ICU (25.2%–100%), followed by neonatal ICU/ward (7.0%–53.6%) and paediatric medical ward (2.7%–33.0%). Surgical site infection was the most common HAIs and accounted for 41.6% of all HAIs (95% CI 23.55–59.80), followed by bloodstream infection (17.07%, 95% CI 11.80–22.33) and respiratory tract infections/pneumonia (17.04%, 95% CI 13.21–20.87). Recent hospitalization (adjusted odds ratio [AOR]: 4.17, 95% CI 1.85–9.41), presence of peripheral vascular catheter (AOR: 2.87, 95% CI 1.54–5.36) and having diabetes mellitus (AOR: 2.46, 95% CI 1.45–4.17) were the strongest predictors of HAIs in Africa. Only 37.9% of HAIs had documented positive microbiological culture result with gram negative bacteria including *Klebsiella pneumoniae*, *Escherichia coli*, *Pseudomonas aeruginosa*, *Acinetobacter baumannii* and *Citrobacter* been the most common microorganisms and accounted for 40%–100% of the pathogens.

**Conclusions:**

The pooled point-prevalence of HAIs in Africa is more than two times higher than the rate reported in developed countries. The prevalence varied between the countries and was highest in the ICU and neonatal ICU/ward. Surgical site infection and bloodstream infection were the most common HAIs reported in African studies. Recent hospitalization, presence of peripheral vascular catheter and having diabetes mellitus were the strongest predictors of HAIs in African studies. Most of the HAIs are preventable with appropriate infection control measures and antimicrobial stewardship. Additional studies are needed especially in the Central African region. Future studies should be designed using standardized protocol and standardized definition to reduce heterogeneity among the studies.

## Background

Healthcare-associated infections (HAIs) are a threat to patient safety during hospitalization. HAIs are associated with significant morbidity, mortality and healthcare costs, and they also impact negatively on patients’ health-related quality of life [1, 2]. In the United State, approximately 2 million HAIs are reported annually with about 90,000 deaths [3]. In Asia, HAIs prolonged hospital stay by 5–21 days and HAIs are associated with mortality ranging from 7% to 46% [2]. In Africa, a higher rate of mortality among inpatients who suffer HAIs has been reported (22.0%) [4]. In addition, HAIs are associated with multidrug resistant pathogens which constitutes a burden to patient’s clinical and economic outcomes [5]. Existing data suggest that the burden of HAIs is higher in low and middle income countries compared to developed countries [6, 7]. In the United States, HAIs affect about 4% of patients admitted to acute care health facilities [8], while 6.5% and 3.9% of patients in acute care hospitals and long-term care facilities in Europe have at least one HAI, respectively [9]. In Asia, it is estimated that 9.0% of hospitalized patients develop at least one HAI with higher incidence reported in the intensive care unit (ICU) [2]. There are limited data describing the burden of HAIs in healthcare facilities across Africa; however, it is estimated that the prevalence of HAIs is much higher than in developed countries [7, 10]. Lack of infection prevention and control program and lack of hand hygiene training and infrastructure are some of the reasons responsible for the high rate of HAIs in Africa [11, 12]. Most HAIs are preventable using evidence-based multifaceted infection control and prevention measures [1, 13]. However, understanding the epidemiology of HAIs is a prerequisite for designing effective infection prevention and control interventions.

Point-prevalence surveys have been used in the US and by the European Centre for Disease Prevention and Control for surveillance of HAIs [8, 9]. There is a lack of surveillance system to monitor HAIs in healthcare facilities across Africa. Previous estimate of HAIs in Africa was reported in a systematic reviewed conducted over 10 years ago [7]. In recent years, several point-prevalence studies conducted in healthcare facilities across Africa have been published. The primary objective of this study is to estimate the pooled point-prevalence and types of HAIs among hospitalized patients in Africa. The secondary objectives are to evaluate the risk factors associated with HAIs and to describe the microorganisms isolated from patients with HAIs in African studies.

## Methods

### Study design

This systematic review and meta-analysis of healthcare-associated infections in Africa was conducted in accordance with the Preferred Reporting Items for Systematic review and Meta-Analysis (PRISMA) statement 2020 [14].

### Eligibility criteria

#### Inclusion criteria

1. Point-prevalence studies conducted among humans in acute care settings in Africa.
2. Studies published between January 2010 and March 2022. The review was limited to studies published from January 2010 to provide estimates of the outcomes based on recent studies. In addition, most point-prevalence surveys conducted in Africa were published from 2010 onward.
3. Studies conducted in all age groups and all inpatient settings.
4. Studies that were published in English language and available as free full-text.

#### Exclusion criteria

1. Longitudinal studies, case-series, and case reports.
2. Point-prevalence survey of healthcare associated infections in a specific patient population such as COVID-19 patients were excluded.
3. Previous systematic reviews and meta-analyses, editorials, letters to editors, commentaries and unpublished articles.

### Information sources

Two electronic databases PubMed and Scopus were used to identify eligible articles. The databases were searched from 01/01/2010 to 04/03/2022 using the search terms described below. In addition, Google Scholar was also searched to find additional studies. The reference lists of selected studies was also examined for eligible articles.

### Search strategy

The keywords “point-prevalence study”, “healthcare associated infections”, and “Africa” with their synonyms were combined using Boolean indicators. The following keywords were used to conduct the search on the electronic databases: point prevalence survey OR point prevalence study OR point-prevalence survey OR point-prevalence study OR point-prevalence OR point prevalence AND healthcare-associated infection OR healthcare-associated infections OR healthcare associated infection OR healthcare associated infections OR hospital-acquired infection OR hospital-acquired infections OR hospital acquired infection OR hospital acquired infections OR nosocomial infection OR nosocomial infections AND Africa.

### Selection process

All the articles identified from the electronic databases were combined and screened to identify and remove duplicates. The titles and abstracts of the non-duplicate articles were screened by two independent reviewers (UA and OA) based on the eligibility criteria. The full-text of the studies that fulfil the eligibility criteria were reviewed for data collection by two reviewers (UA and OA). Disagreement between the reviewers was resolved through consensus.

### Data collection process

The selected studies were reviewed and the data were collected using a predesigned data collection form. Data collection was conducted by two independent reviewers (UA and OA). Consensus was used to address any disagreements between the reviewers.

### Data items

The following information was extracted from the selected studies: first author’s name and year of publication, country involved, study setting/number of centre(s), study design, study period, number of patients involved, PPS protocol used (ECDC, CDC or as defined by the authors), overall prevalence of HAIs, types of HAIs and their prevalence, the risk factors associated with HAIs and their odds ratio, and the microorganisms that caused HAIs among the patients.

### Quality assessment

The methodological quality of the selected studies was assessed by two independent reviewers (UA and OA) using the Newcastle–Ottawa scale (NOS) [15]. The NOS consists of three sections including selection, comparability, and outcomes. Disagreements between the reviewers were resolved through consensus.

### Outcome assessment and effect measures

The primary outcome was the overall point-prevalence of HAIs among hospitalized patients in Africa. HAI was defined as an infection diagnosed among hospitalized patients which was not present or incubating at the time of admission. This definition is based on the Centers for Disease Control and Prevention (CDC) [16] and the European Centres for Disease Prevention and Control (ECDC) guidelines [17]. Secondary outcomes include types of HAIs and their prevalence among hospitalized patients as defined by the guidelines [16, 17]. Other secondary outcomes include the risk factors associated with HAIs and the microorganisms that caused HAIs among hospitalized patients. The primary outcome was presented as frequency and percentage. The types of HAIs were presented as frequency and percentage. The risk factors associated with HAI were presented as adjusted odds ratio, while the microorganisms were retrieved as frequency and percentage.

### Synthesis methods

The data were synthesized using both qualitative and quantitative methods. Quantitative synthesis was conducted using RevMan 5.4 software. The overall point-prevalence of HAIs was evaluated as the number of patients with HAI on the day of the survey as a proportion of all hospitalized patients on the day of the survey. The pooled estimate was determined using random-effects meta-analysis. The selected studies were from different countries and the patient population varied. Therefore, our objective was to estimate the mean of a distribution of effects. The results were described using forest plots. Heterogeneity was examined using the Higgins *I*^2^ statistic, with < 40% considered as low heterogeneity, 30–60% moderate heterogeneity, 50–90% substantial heterogeneity and 75–100% considerable heterogeneity [18]. To provide pooled risk estimates for the factors associated with HAIs, a meta-regression analysis was performed for risk factors found to be significant in at least two of the selected studies.

## Results

### Study selection

**Fig. 1:**
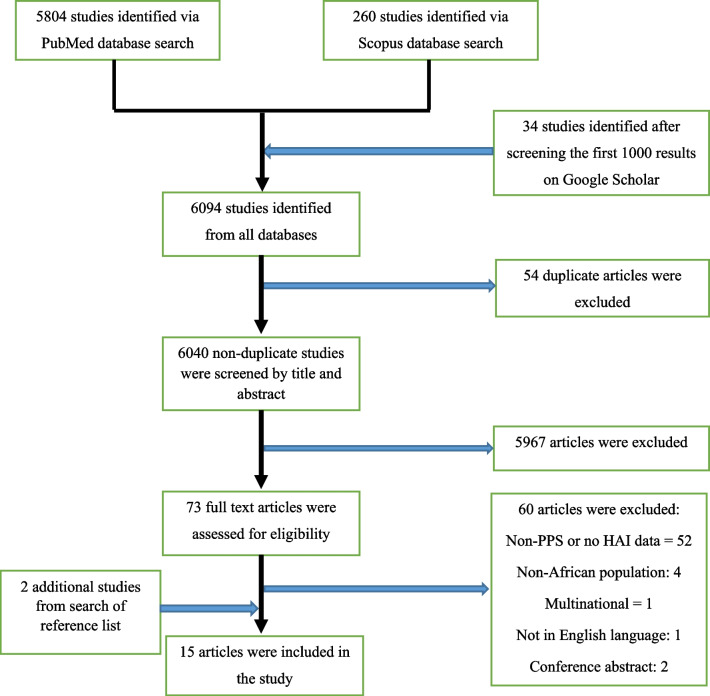
Flow chart of article screening and selection

A total of 6094 articles were identified from the electronic databases and 54 duplicates were removed. After screening the title and abstracts of the non-duplicate articles, 5967 articles were excluded. The full-text of 73 articles was reviewed and 13 eligible studies were selected. Two additional studies were found after checking the reference list of selected studies. Overall, 15 studies were included in this systematic review, while 11 studies were selected for the meta-analysis. Figure 1 shows the article screening and selection process.

### Characteristics of selected studies

Six (40%) studies were conducted in North Africa (four from Tunisia and two from Morocco). West Africa (one each from Nigeria, Ghana and Benin Republic) and South Africa (two and one from South Africa and Botswana, respectively) had three studies each. In East Africa, one study was conducted in Ethiopia, Uganda and Malawi. Most studies (*n* = 9, 60.0%) involved multiple centres and 12 studies (80.0%) were hospital-wide point-prevalence studies (including multiple units in the hospital). One study each involved patients in a single unit/ward including ICU, surgery and paediatric/neonatal unit. Overall, a total of 11,272 hospitalized patients were included in the selected studies and the patient population ranged between 103 and 2107 patients. Table 1 summarizes the characteristics of the selected studies.

**Table 1:** Characteristics of the studies included in this review

S/no.	Author and year	Country and continent	Study setting/no. of centres	Guideline used	Period of the study	Number of participants	Prevalence of HAIs	Prevalence of different types of HAIs	Prevalence of different HAIs among total HAIs	Prevalence of HAIs in speciality/ward
1	Labi et al., 2019 [23]	Ghana	Multicentre (10 hospitals)	ECDC	September to December 2016	2107	172/2107 (8.2%)	SSI: 2.8% BSI: 1.7% UTI: 1.6% RTI: 1.4%	SSI: 32.9% BSI: 19.5% UTI: 18.5% RTI: 16.3% DAI: 7.1%	Surgical: 11.2 Paediatrics: 8.0 Medical: 7.5 OBG: 6.4 Psychiatry: 4.5
2	Ketata et al., 2021 [29]	Tunisia	Bicentric (2 hospitals)	CDC	February 2019	898	65/898 (7.24%)	SSI: 1.11% BSI: 1.22% UTI: 2.56% RTI: 2.0%	SSI: 12.3% BSI: 13.6% UTI: 28.4% RTI: 22.2%	NA
3	Bunduki et al., 2021 [32]	Malawi	Single centre (department of surgery only)	ECDC	June 2020	105	12/105 (11.4%)	NA	SSI: 33.3% BSI: 25.0% UTI: 33.3% Bone/Joint infection: 8.3%	Surgery: 11.4%
4	Usman [22]	Nigeria	Multicentre (3 hospitals)	ECDC	April to May 2019	321	46/321 (14.3%)	SSI: 5.0% BSI: 5.9% UTI: 2.56% Pneumonia: 1.9% GI: 1.6% EENTI: 0.6% SSTI: 0.3%	SSI: 32.0% BSI: 38.0% UTI: 18.5% Pneumonia: 12.0% GI: 10.0% EENTI: 6.0% SSTI: 2.0% DAI: 2.5%	Pediatric: 2.7% Neonatal: 53.6% Medical: 9.2% Surgical: 10.1% OBG: 10.0% Pediatric surgery: 14.6%
5	Chiguer et al., 2018 [28]	Morocco	Single centre	ECDC	June–July 2017	207	46/207 (22.2%)	NA	SSI: 15.21% Systemic infection: 8.7% UTI: 17.39% LRTI: 8.7% GI: 10.0% EENTI: 2.17% SSTI: 10.87%	NA
6	Yallew et al., 2016 [31]	Ethiopia	Multicentre (2 hospitals) Repeated point-prevalence	CDC	March to July 2015	908	135/908 (14.9%)	SSI: 7.59% BSI: 2.09% UTI: 0.99% Pneumonia: 2.75% GI: 0.55% SSTI: 0.55%	SSI: 51.1% BSI: 14.1% UTI: 6.7% Pneumonia: 18.5% GI: 3.7% SSTI: 3.7%	NA
7	Ahoyo et al., 2014 [4]	Benin Republic	Multicentre (39 hospitals)	CDC	October 2012	3130	597/3130 (19.1%)	NA	SSI: 19.2% IVCI: 27% BSI: 1.5% UTI: 37.5% LRTI: 11.7%	
8	Jamoussi et al., 2018 [27]	Tunisia	Multicentre (15 ICUs)	NA	September 2017	103	32/103 (25.2%)	Pneumonia: 18.4% CR infection: 4.8% BSI: 2.9% UTI: 2.9% SSTI: 0.9% Peritonitis: 0.9%	Pneumonia: 59% CR infection: 15%	ICU: 25.2%
9	Mpinda-Joseph et al., 2019 [21]	Botswana	Single centre	CDC	November 2017	347	47/347 (13.54%)	NA	SSI: 23.4% Ventilator associated infection: 17% Debitucus ulcer: 10.6% LCBSI: 8.5% UTI: 6.4%	ICU: 100% Nephrology: 50% SCBU: 41.9% Pediatric medical: 33.3%
10	Nair et al., 2018 [20]	South Africa	Single centre	CDC	February to March 2016	326	25/326 (7.67%)	BSI: 0.92% SSI: 4.6% LRTI: 0.92% UTI: 1.53%	BSI: 11.5% SSI: 57.7% LRTI: 11.5% UTI: 19.2%	General surgery: 7.69% Internal medicine: 4.11% Paediatrics: 6.12% Orthopaedics: 15.56% OBG: 11.11% Ophthalmology: 0%
11	Olivier et al., 2018 [19]	South Africa	Multicentre (2 hospitals—pediatric and neonatal wards only)	CDC	December 2016	151	15/151 (9.9%)	Pneumonia: 3.3% BSI: 1.9% UTI: 1.9% SSI: 0.6%	Pneumonia: 33.3% BSI: 20.0% UTI: 20%	Neonatal ward: 7.0% Paediatric: 11.7%
12	Ayed et al., 2019 [26]	Tunisia	Multicentre (2 hospitals)	CDC	July 2017	752	76/752 (10.0%)	RTI: 4.0% UTI: 1.86% SSI: 1.6% EENTI: 0.8% BSI: 0.66% GI: 0.53% SSTI: 0.4%	RTI: 36.6% UTI: 17.1% SSI: 14.6% EENTI: 7.3% BSI: 6.1% GI: 4.9% SSTI: 3.6%	NA
13	Mahjoub et al., 2015 [25]	Tunisia	Single centre	CDC	2012	312	39/312 (12.5%)	NA	PVC: 42.2% RTI: 15.6% EENTI: 13.4%	Medical: 22 (56.4%) Surgical: 17 (43.66%)
14	Razine et al., 2012 [24]	Morocco	Multicentre	CDC	January 13–15 2010	1195	116/1195 (9.7%)	NA	UTI: 35% SSI: 29.3% LRTI: 10.6% BSI: 8.1% SSTI: 5.7%	ICU: 34.5% OBG: 12.1% Surgery: 13.5 Pediatric: 9.7 Medical: 4.5% Pediatric surgery: 1.5%
15	Greco and Magombe, 2011 [30]	Uganda	Single centre	WHO	February 2010	410	115/410 (28.0%)	NA	NA	NA

*HAI* healthcare-associated infections, *CDC* Centers for Disease Control and Prevention, *ECDC* European Centre for Disease Prevention and Control, *WHO* World Health Organisation, *BSI* bloodstream infection, *SSI* surgical site infection, *UTI* urinary tract infection, *LRTI* lower respiratory tract infection, *RTI* respiratory tract infection, *GI* gastrointestinal infection, *SSTI* skin and soft tissue infection, *EENTI* ear, eye, nose and throat infection, *URTI* upper respiratory tract infection, *OR* odds ratio, *AOR* adjusted odds ratio, *CI* confidence interval, *NA* not available

### Quality Assessment

Overall, most of the studies (*n* = 12; 80%) have good quality. Two of the studies were found to have moderate quality, while one study had low quality. The sample were representative in 13 (86.7%) studies. There was low risk of bias in the assessment of outcomes and statistical test domains with 12 (80%) studies having good quality rating. Nine studies had a total score of 8 points, three studies had 7 points, while three studies had less than 6 points. Table 2 shows the quality assessment of the selected studies.

**Table 2:** Assessment of risk of bias for the studies included in the review

Author name and year	Selection				Comparability	Outcomes		Quality score	Quality scale
	Representatives of sample	Sample size	Non-respondents	Ascertainment of exposure	Based on design and analysis	Assessment of outcomes	Statistical test		
Labi et al., 2019 [23]	*	*	NA	**	*	**	*	8	Good
Ketata et al., 2021 [29]	*	*	NA	**	*	**	*	8	Good
Bunduki et al., 2021 [32]	–	*	NA	**	*	**	*	7	Good
Abubakar Usman [22]	*	*	NA	**	*	**	*	8	Good
Chiguer et al., 2018 [28]	*	*	NA	**	*	**	*	8	Good
Yallew et al., 2016 [31]	*	*	NA	**	*	**	*	8	Good
Ahoyo et al., 2014 [4]	*	*	NA	**	–	–	*	5	Moderate
Jamoussi et al., 2018 [27]	*	–	NA	*	*	–	*	4	Moderate
Mpinda-Joseph et al., 2019 [21]	*	*	NA	**	*	**	–	7	Good
Nair et al., 2018 [20]	*	*	NA	**	*	**	*	8	Good
Olivier et al., 2018 [19]	–	*	NA	**	–	–	–	2	Low
Ayed et al., 2019 [26]	*	*	NA	**	*	**	*	8	Good
Mahjoub et al., 2015 [25]	*	*	NA	**	*	**	–	7	Good
Razine et al., 2012 [24]	*	*	NA	**	*	**	*	8	Good
Greco and Magombe, 2011 [30]	*	*	NA	**	*	**	*	8	Good

*NA* not applicable

### Qualitative summary of results

#### Prevalence and distribution of HAIs among hospitalized patients in Africa

Fifteen studies were included in the qualitative summary of the prevalence of HAIs among hospitalized patients in Africa. Overall, the prevalence of HAIs ranged between 7.24% and 28% [4, 19–32], and the prevalence varied between the regions in Africa. The prevalence of HAIs in East, North, West and South Africa region ranged between 11.4–28.0% [30–32], 7.2–25.2% [24–29], 8.2–19.1% [4, 22, 23], and 7.6–13.5% [19–21], respectively. The highest prevalence of HAIs was reported in Uganda (28.0%) [30], followed by Tunisia (25.2%) [27], Morocco (22.2%) [28], Benin Republic (19.1%) [4] and Ethiopia (14.9%) [31]. The lowest prevalence of HAIs was reported in another Tunisian study (7.2%) [29], followed by South Africa (7.6%) [20] and Ghana (8.2%) [23]. The prevalence of HAIs varied between the wards with the highest rate reported in the ICU (25.2–100%) [21, 27, 30], followed by special care baby unit/neonatal ward (7.0–53.6%) [19, 21, 22], paediatric medical ward (2.7–33.0%) [19–22, 30], and surgical ward (7.6–13.5%) [20, 22, 23, 30, 32]. The rate of HAIs in the obstetrics and gynaecology, and medical ward was (6.4–12.1%) [20, 22, 23, 30] and (4.11–9.2%) [20, 22, 23, 30], respectively. Several types of HAIs were described in the studies included in this review. The most common types of HAIs reported include respiratory tract infections (8.7–59%) [4, 19, 20, 22, 23, 25–31], surgical site infections (12.3–57.7%) [4, 20–23, 26, 28–32], urinary tract infection (6.4–37.5%) [4, 19–23, 26, 28–32] and bloodstream infections (1.5–38.0%) [4, 19, 20, 22, 23, 26, 29–32]. Other infections included ear, eye, nose and throat infections (2.1–13.4%) [22, 25, 26, 28], gastrointestinal infections (3.7–10.0%) [22, 26, 28, 31] and skin and soft tissue infections (2.0–10.8%) [22, 26, 28, 30, 31]. Table 1 shows the prevalence and types of HAIs reported in the selected studies.

### Quantitative summary of results

#### Meta-analysis of prevalence of HAIs in Africa

**Fig. 2:**
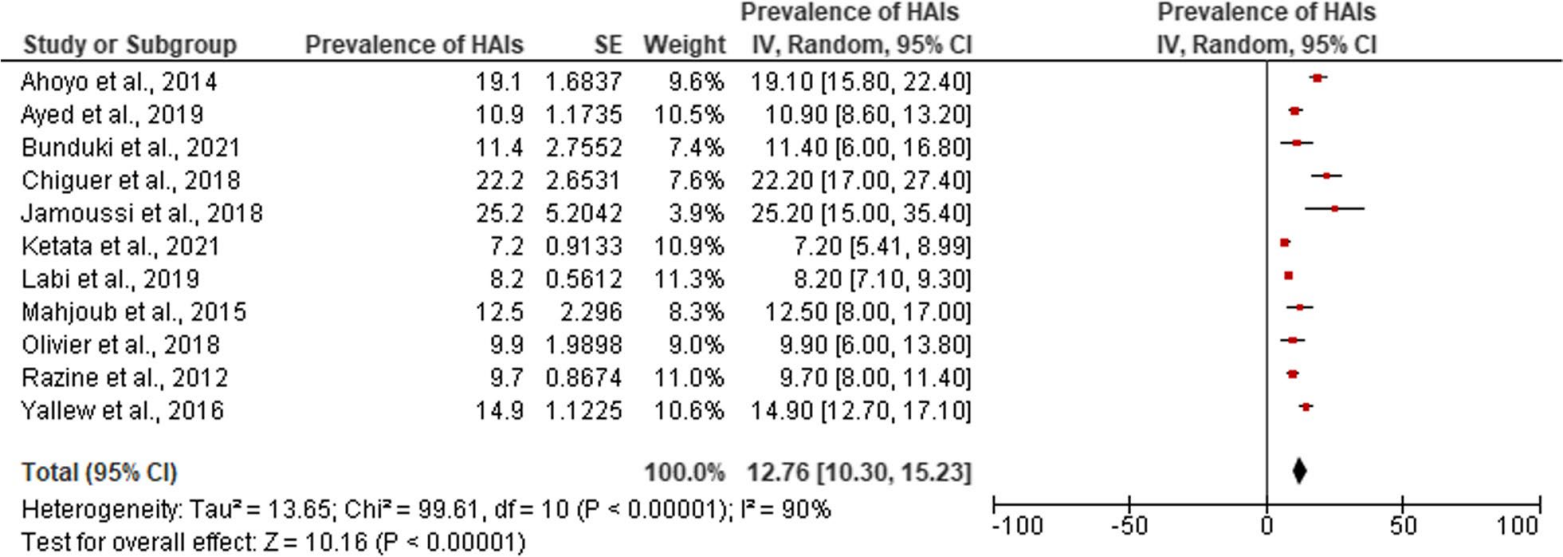
Forest plot of the prevalence of HAIs among hospitalized patients in Africa

**Fig. 3:**
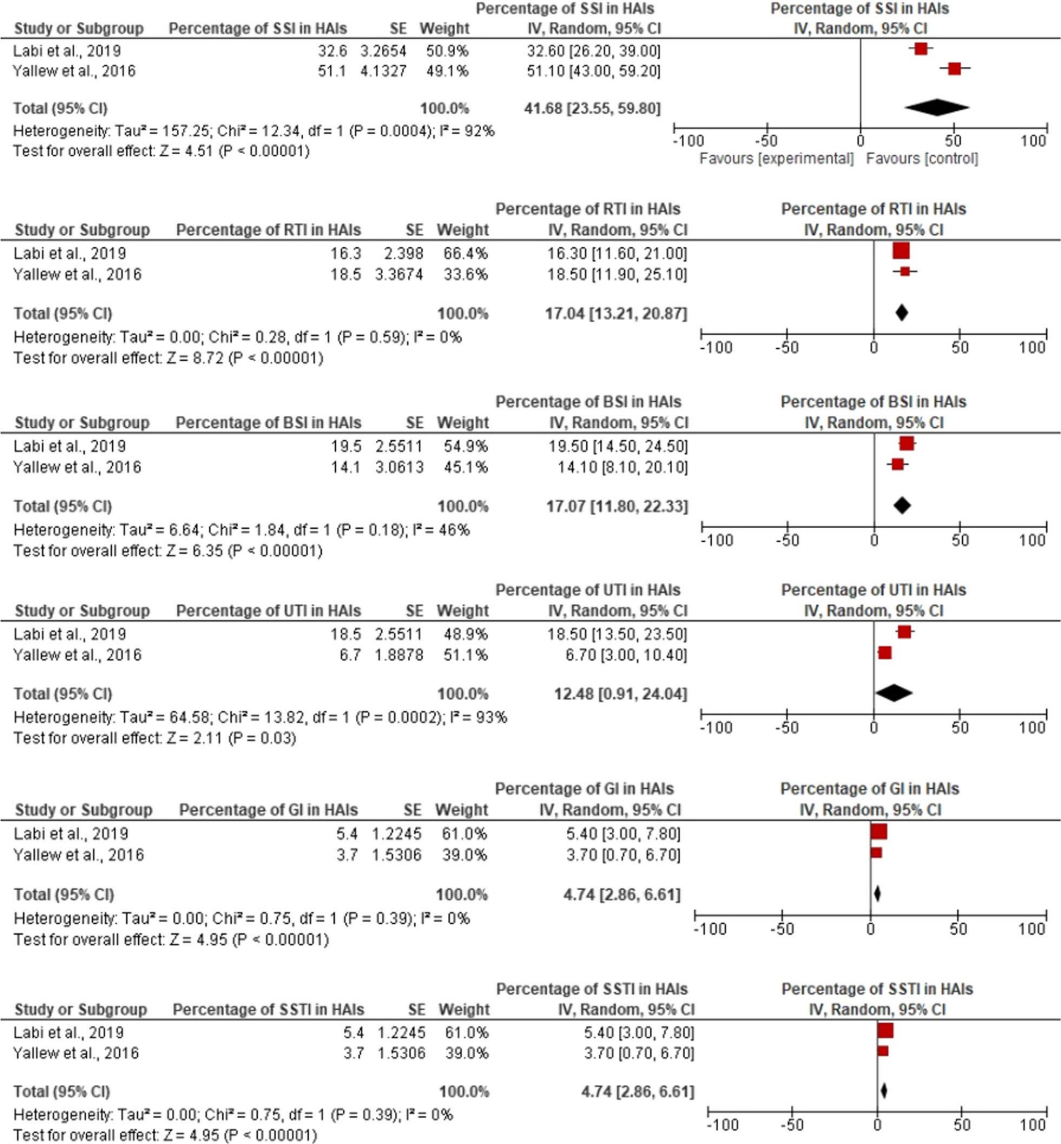
Forest plots for the percentage of distributions of HAIs reported in African studies

Of the fifteen studies, eleven studies fulfilled the criteria and were included in the meta-analysis [4, 19, 23, 25–32]. The pooled point-prevalence of HAIs in Africa was 12.76% (95% confidence interval 10.30–15.23). A high degree of heterogeneity was observed (*I*^2^ = 90.0%; *P* < 0.0001) and the overall effect size was 10.16 (*p* < 0.0001). Figure 2 illustrates the forest plot for the pooled point-prevalence of HAIs among hospitalized patients in Africa. Surgical site infections were the most common HAIs in Africa and accounted for 41.6% of all HAI (95% CI 23.55–59.80), followed by bloodstream infection which represented 17.07% (95% CI 11.80–22.33) and respiratory tract infections/pneumonia with 17.04% (95% CI 13.21–20.87). Figure 3 summarises the results of the meta-analysis for the percentage of distributions of HAIs reported in African studies.

### Risk factors associated with HAIs in Africa

There were numerous risk factors associated with HAIs reported in Africa. Meta-regression analysis showed that recent hospitalization (adjusted odds ratio [AOR]: 4.17, 95% confidence interval [CI] 1.85–9.41, *p* < 0.001) was the major risk factor associated with HAIs in Africa, followed by the presence of peripheral vascular catheter (AOR: 2.87, 95% CI 1.54–5.36, *p* < 0.001) and having diabetes mellitus (AOR: 2.46, 95% CI 1.45–4.17, *p* < 0.001). Other significant risk factors include admission to surgical ward (AOR: 2.19, 95% CI 1.41–3.42, *p* < 0.001) and ultimately fatal McCabe score (AOR: 2.09, 95% CI 1.35–3.24, *p* < 0.001). Table 3 depicts the results of the meta-regression analysis for the factors associated with HAIs reported in healthcare facilities in Africa.

**Table 3:** Meta-regression analysis of risk factors associated with HAIs in Africa

Risk factor	Author/year	Pooled AOR (95% CI)	Overall effect size	*P* value	*I*^2^ (%)
Duration of hospital stay	Ahoyo et al., 2014 [4] Chiguer et al., 2018 [28] Labi et al., 2019 [23]	2.85 (0.92–3.72)	1.81	0.07	98
Surgery during admission	Chiguer et al., 2018 [28] Labi et al., 2019 [23]	3.38 (0.83–13.88)	1.69	0.09	86
Urinary catheter	Labi et al., 2019 [23] Razine et al., 2012 [24]	4.07 (0.75–21.99)	1.63	0.10	95
Presence of peripheral vascular catheter	Ayed et al., 2019 [26] Ketata et al., 2021 [29] Labi et al., 2019 [23] Mahjoub et al., 2015 [25]	**2.87 (1.54–5.36)**	**3.31**	**0.0009**	44
Admission into surgical department/ward	Labi et al., 2019 [23] Yallew et al., 2016 [31]	**2.19 (1.41–3.42)**	**3.48**	**0.0005**	52
McCabe score (ultimately fatal disease)	Chiguer et al., 2018 [28] Ketata et al., 2021 [29] Razine et al., 2012 [24]	**2.09 (1.35–3.24)**	**3.32**	**0.0009**	0
Diabetes mellitus	Ayed et al., 2019 [26] Ketata et al., 2021 [29]	**2.46 (1.45–4.17)**	**3.35**	**0.0008**	0
Recent hospitalization	Jamoussi et al., 2018 [27] Ketata et al., 2021 [29]	**4.17 (1.85–9.41)**	**3.44**	**0.0006**	0

Bold values indicate statistical significance

### Pathogens involved in HAIs

Overall, only 37.9% of HAIs had documented positive microbiological culture result and the pathogens identified include bacteria and fungi. Gram negative bacteria including *Klebsiella pneumoniae*, *Escherichia coli*, *Pseudomonas aeruginosa*, *Acinetobacter baumannii* and *Citrobacter* were the most common microorganisms and accounted for 40–100% of the pathogens. This was followed by gram positive bacteria such as *Staphylococcus aureus*, *enterococci* and *streptococci*, and fungi such as *Candida albicans* and *Aspergillus fumigatus* which account for 21.7–50% and 1.8–21.7%, respectively. Table 4 shows the distribution of the pathogens identified as causes of HAIs in African studies.

**Table 4:** Distribution of microorganisms cultured in HAIs in Africa

S/no.	Author and year	Microorganisms associated with HAIs	Percentage of HAIs with documented microbiological culture
1	Labi et al., 2019 [23]	Gram negative: 16	23/184 (12.5%)
		Gram positive: 7	
2	Ketata et al., 2021 [29]	Gram negative	53 (65.4%)
		a. *K. pneumoniae*: 12	
		b. *Escherichia coli*: 11	
		c. *Acinetobacter baumanni*: 5	
		d. *Pseudomonas aeruginosa*: 4	
		Gram positive	
		a. *Staphylococcus aureus*: 10	
		b. *Enterococcus faecalis*: 4	
		c. *Streptococcus pneumoniae*: 1	
		*Candida albican*: 1	
3	Abubakar [22]	Gram negative: 2 (50%)	4/46 (8.7%)
		a. *E. coli*: 1	
		b. *Proteus* spp.: 1	
		Gram positive: 2 (50%)	
		a. *S. aureus*: 1	
		b. *Methicillin-resistant S. aureus (MRSA)*: 1	
5	Yallew et al., 2016 [31]	Gram negative: (…%)	
		a. *Klebsiella* spp. (22.44%)	
		b. *P. aeruginosa* (18.36%)	
		c. *E. coli* (16.32%)	
		d. *Enterobacter* spp. (12.24%)	
		e. *Proteus* spp. (6.12%)	
		f. *Citrobacter* spp. (6.12%)	
		g. *K. pneumoniae* (4.08%)	
		h. *Acinetobacter* spp. (4.08%)	
		i. *Serratia* spp. (2.04%)	
		Gram positive	
		a. *S. aureus* (20.40%)	
		b. *S. pneumoniae* (10.20%)	
6	Ahoyo et al., 2014 [4]	Gram negative	
		a. *E. coli*: 22.7%	
		b. *P. aeruginosa*: 11.3%	
		c. *Salmonella* spp.: 4.2%	
		d. *Citrobacter* spp.: 2.7%	
		e. *A. baumanii*: 1%	
		f. Other GNR (99) 10.2	
		Gram positive	
		a. *S. aureus*: 27.9%	
		b. *Enterococci*: 10.5%	
		c. CNS (47) 4.9	
		*Candida* spp. (15) 1.5	
		Undetermined (30) 3.1	
7	Jamoussi et al., 2018 [27]	Gram negative	21/32 (66%)
		a. *P. aeroginosa*: 10 cases	
		b. *A. baumanii*: 2 cases	
		c. *K. pneumoniae*: 4 cases	
		d. *P. mirabilis*: 2 cases	
		e. *Citrobacter freundii*: one case	
8	Nair et al., 2018 [20]	Gram negative	
		a. *K. pneumoniae*: 4 cases	
		b. *P. aeruginosa*: 2 cases	
		c. *A. baumannii*: 1 case	
		d. *Enterobacter cloacae*: 1 case	
		Gram positive	
		a. *S. aureus*: 2 cases	
		*Candida albicans*: 1 case	
9	Olivier et al., 2018 [19]	Gram negative	6/15 (40%)
		a. *E. coli* = 2	
		Gram positive	
		b. Group B *Streptococci*: *n* = 2	
		c. *S. aureus: n* = 1, *Candida albicans*: *n* = 1	
10	Ayed et al., 2019 [26]	Gram negative	52/82 (63.4%)
		a. *E. coli*: 5 (9.6%)	
		b. *K. pneumoniae*: 5 (9.6%)	
		c. *A. baumannii*: 6 (11.5%)	
		d. *P. aeruginosa*: 8 (15.4%)	
		e. *S. pneumoniae*: 4 (7.7%)	
		f. *N. meningitidis*: 2 (3.8%)	
		Gram positive	
		a. *E. faecalis*: 3 (5.8%)	
		b. *S. aureus*: 3 (5.8%)	
		Fungi	
		a. *C. albican*: 4 (7.7%)	
		b. *A. fumigatus*: 6 (11.5%)	
11	Mahjoub et al., 2015 [25]	Gram negative: 4	6/45 (13.3%)
12	Razine et al., 2012 [24]	Gram negative	75/123 (61%)
		a. *E. coli* (14.7%)	
		b. *K pneumoniae* (14.7%)	
		Gram positive	
		a. *Staphylococcus* (18.7%)	

## Discussion

This study evaluated the prevalence and types of HAIs, and pathogens implicated in those infections among hospitalized patients in Africa. The study found that there are limited studies that reported the rate of HAIs among hospitalized patients, particularly in the Central African region, where there was limited number of studies. The studies used different guidelines to conduct the point-prevalence studies reflecting the lack of an African protocol for conducting point-prevalence of HAIs in African hospitals. Therefore, the development and validation of a protocol for conducting point-prevalence studies in African hospitals is recommended and this can be spearheaded by the African Centres for Control and Prevention. Most of the studies included in this review had good quality and minimal risk of bias. The pooled prevalence of HAIs in Africa was about 12.8% and this is higher than the prevalence reported in Asia (9.0%) [2], Europe (6.5%) [9] and the United States (4.0%) [8]. However, the rate of HAIs is lower than the 15.5% reported in developing countries [6] and 16.9% reported in Ethiopia [33, 34]. Higher rate of HAIs in Africa could be explained by poor infection control and prevention practices in the continent due to limited infection prevention and control capacity [35]. Previous studies have shown poor rate of adherence to hand hygiene among healthcare workers in sub-Saharan Africa due to lack of knowledge and training, heavy workload and lack of infrastructure [11]. Therefore, strategies to improve compliance with hand hygiene among healthcare workers including training, provision of soap and water as well as alcohol-based hand rub are recommended [12]. The improvement in hand and environmental hygiene during the COVID-19 pandemic may have an impact on the rate of HAIs in Africa but there is lack of evidence to demonstrate changes in the rate of HAIs during the pandemic.

The prevalence of HAIs seems to be higher in the East African region and lower in the South African region. The reasons for this variations are unclear but it could be attributed to the use of different methods and tools to define HAIs among the studies. Further studies are required to confirm these observations. Existing evidence has shown that patients admitted into the ICU have higher risk of developing HAIs [8]. The current review confirms these data, with the finding of higher rate of HAIs in the ICU. This is in consonance with the finding of a meta-analysis in Ethiopia [33]. The result also revealed high rate of HAIs in neonatal and paediatric wards which is consistent with the findings of a previous systematic review and meta-analysis of HAIs in Ethiopia [10], and in developing countries [6]. Therefore, continuous HAIs surveillance, implementation of hand and environmental hygiene, and training of healthcare workers on infection prevention and control measures are recommended to reduce the burden of HAIs in these wards/units. Previous studies have revealed that surgical site infection is the most common HAI reported in healthcare facilities in Africa [7, 34, 36]. In addition, surgical site infection is the most common HAI reported in acute care facilities the US [8]. The current study confirmed these data with surgical site infection accounting for about 4 in every 10 HAIs in Africa. The high rate of surgical site infection could be attributed to lack of adequate infection control before, during and after surgery coupled with inappropriate use of surgical antimicrobial prophylaxis. Available data revealed low compliance with the timing and duration of antimicrobial prophylaxis [37–39]. Therefore, antimicrobial stewardship and infection prevention and control are recommended to reduce the burden of surgical site infections in Africa [40]. Bloodstream infection and respiratory tract infections including pneumonia were the second and third most common HAIs in Africa. These infections are mostly device-associated and are preventable with appropriate infection prevention and control measures. Hand and environmental hygiene as well as injection safety practices should be encouraged in African healthcare facilities. The COVID-19 pandemic has caused significant disruption in healthcare systems including infection prevention and control [41–44]. The diversion of traditional infection prevention and control resources and measures, such as active surveillance; screening programme to detect colonization, and isolation of patients with multidrug resistant infections; to the management of the pandemic may increase the prevalence of HAIs [42–45]. In contrast, evidence has shown that healthcare workers had good infection control practices during the pandemic and this will reduce the transmission of HAIs [46]. However, there is a lack of data to describe the impact of COVID-19 pandemic on the prevalence of HAIs in healthcare facilities across Africa.

Several risk factors have been reportedly associated with HAIs in African studies and our analysis revealed that those with history of recent hospitalization had the highest risk of HAIs, followed by the presence of peripheral vascular catheter and those with diabetes mellitus. Frequent hospitalization is more common in patients with multiple comorbidities or those with complicated chronic illnesses which predispose such patients to an increased risk of HAIs and colonization or infection with multidrug resistant pathogens. The use of peripheral vascular catheter increases the risk of bloodstream infection among hospitalized patients and this explains the association between HAIs and the presence of peripheral catheter in our study. Patients with diabetes mellitus have an increased risk of common infections compared to those without diabetes mellitus [47]. The current review confirmed these data with the finding of higher risk of HAIs among diabetes mellitus patients in Africa. This finding is consistent with a previous meta-analysis in Ethiopia that showed a significant association between HAIs and underlying non-communicable disease [33]. Therefore, efforts to achieve and maintain glycaemic control among diabetes mellitus patients during hospitalization is recommended in addition to improved hand and environmental hygiene practices. Other factors significantly associated with HAIs in Africa include admission into surgical ward and underlying ultimately fatal McCabe score.

The current review revealed that gram negative pathogens such as *E. coli*, *K. pneumoniae*, *P. aeruginosa* and *A. baumannii* were the most common causative organisms associated with HAIs. This is consistent with findings of a previous systematic reviews [2, 6]. Other pathogens include gram positive bacteria and fungi, such as *C. albicans* and *A. fumigatus*. The current review has a number of limitations that warrant caution in interpreting the results. Firstly, there was limited number of point-prevalence studies conducted in Central African region and limited number of studies from the other regions. This may potentially affect the generalizability of the results. Secondly, the degree of heterogeneity was high and this was lower than the heterogeneity reported in a previous systematic review and meta-analysis [6]. Differences in study protocol, data collection method, study period, and definition of HAIs between the studies may explain the high degree of heterogeneity. Future studies should develop and validate an African protocol for point-prevalence survey of HAIs in African hospitals. This will bring about consistency in the design, data collection and description of HAIs in Africa. The African CDC should develop a standardise protocol for conducting point-prevalence studies for HAIs in Africa to ensure consistency between studies. Thirdly, the reporting of point-prevalence studies of HAIs in Africa need to improve as many studies did not describe the confidence interval for the point-prevalence of HAIs in different wards/units and the confidence interval for the different types of HAIs. This made it difficult to perform a detailed meta-analysis. Studies describing the impact of HAIs on morbidity, mortality, quality of life and the economic burden of HAIs in Africa are recommended.

## Conclusions

The pooled point-prevalence of HAIs in Africa is relatively high compared to the other continents. The point-prevalence of HAIs in Africa is about two times higher than the rate reported in developed countries. The point-prevalence of HAIs is higher in ICU and neonatal wards compared to the other wards. Surgical site infections and bloodstream infection were the most common HAIs reported in Africa. Recent hospitalization, presence of peripheral vascular catheter and having diabetes mellitus were the strongest risk factors associated with HAIs in Africa. Gram negative bacteria were the major causative pathogens associated with HAIs. Infection prevention and control measures and antimicrobial stewardship are recommended to reduce the burden of HAIs among hospitalized patients in Africa.

## Data Availability

All data generated or analyzed during this study are included in this published article.

## Abbreviations

HAI: Healthcare-associated infections
CDC: Centers for Disease Control and Prevention
ECDC: European Centre for Disease Prevention and Control
WHO: World Health Organisation
BSI: Bloodstream infection
SSI: Surgical site infection
UTI: Urinary tract infection
LRTI: Lower respiratory tract infection
RTI: Respiratory tract infection
GI: Gastrointestinal infection
SSTI: Skin and soft tissue infection
EENTI: Ear, eye, nose and throat infection
URTI: Upper respiratory tract infection
OR: Odds ratio
AOR: Adjusted odds ratio
CI: Confidence interval

## References

[1] Storr J, Twyman A, Zingg W, Damani N, Kilpatrick C, Reilly J, Price L, Egger M, Grayson ML, Kelley E, Allegranzi B (2017). Core components for effective infection prevention and control programmes: new WHO evidence-based recommendations. Antimicrob Resist Infect Control.

[2] Ling ML, Apisarnthanarak A, Madriaga G (2015). The burden of healthcare-associated infections in Southeast Asia: a systematic literature review and meta-analysis. Clin Infect Dis.

[3] Stone PW (2009). Economic burden of healthcare-associated infections: an American perspective. Expert Rev Pharmacoecon Outcomes Res.

[4] Ahoyo TA, Bankolé HS, Adéoti FM, Gbohoun AA, Assavèdo S, Amoussou-Guénou M, Kindé-Gazard DA, Pittet D (2014). Prevalence of nosocomial infections and anti-infective therapy in Benin: results of the first nationwide survey in 2012. Antimicrob Resist Infect Control.

[5] Serra-Burriel M, Keys M, Campillo-Artero C, Agodi A, Barchitta M, Gikas A, Palos C, Lopez-Casasnovas G (2020). Impact of multi-drug resistant bacteria on economic and clinical outcomes of healthcare-associated infections in adults: systematic review and meta-analysis. PLoS ONE.

[6] Allegranzi B, Nejad SB, Combescure C, Graafmans W, Attar H, Donaldson L, Pittet D (2011). Burden of endemic health-care-associated infection in developing countries: systematic review and meta-analysis. Lancet.

[7] Nejad SB, Allegranzi B, Syed SB, Ellis B, Pittet D (2011). Health-care-associated infection in Africa: a systematic review. Bull World Health Organ.

[8] Magill SS, Edwards JR, Bamberg W, Beldavs ZG, Dumyati G, Kainer MA, Lynfield R, Maloney M, McAllister-Hollod L, Nadle J, Ray SM (2014). Multistate point-prevalence survey of health care–associated infections. N Engl J Med.

[9] Suetens C, Latour K, Kärki T, Ricchizzi E, Kinross P, Moro ML, Jans B, Hopkins S, Hansen S, Lyytikäinen O, Reilly J (2018). Prevalence of healthcare-associated infections, estimated incidence and composite antimicrobial resistance index in acute care hospitals and long-term care facilities: results from two European point prevalence surveys, 2016 to 2017. Eurosurveillance.

[10] Irek EO, Amupitan AA, Aboderin AO, Obadare TO (2018). A systematic review of healthcare-associated infections in Africa: an antimicrobial resistance perspective. Afr J Lab Med.

[11] Ataiyero Y, Dyson J, Graham M (2019). Barriers to hand hygiene practices among health care workers in sub-Saharan African countries: a narrative review. Am J Infect Control.

[12] Holmen IC, Seneza C, Nyiranzayisaba B, Nyiringabo V, Bienfait M, Safdar N (2016). Improving hand hygiene practices in a rural hospital in sub-Saharan Africa. Infect Control Hosp Epidemiol.

[13] Schreiber PW, Sax H, Wolfensberger A, Clack L, Kuster SP (2018). The preventable proportion of healthcare-associated infections 2005–2016: systematic review and meta-analysis. Infect Control Hosp Epidemiol.

[14] Page MJ, Moher D, Bossuyt PM, Boutron I, Hoffmann TC, Mulrow CD, Shamseer L, Tetzlaff JM, Akl EA, Brennan SE, Chou R (2021). PRISMA 2020 explanation and elaboration: updated guidance and exemplars for reporting systematic reviews. BMJ.

[15] Wells GA, Shea B, O’Connell D, Peterson J, Welch V, Losos M, Tugwell P. The Newcastle–Ottawa Scale (NOS) for assessing the quality of nonrandomised studies in meta-analyses.

[16] Garner JS, Jarvis WR, Emori TG, Horan TC, Hughes JM (1988). CDC definitions for nosocomial infections, 1988. Am J Infect Control.

[17] European Center for Disease Prevention and Control (2016). Point prevalence survey of healthcare associated infections and antimicrobial use in European acute care hospitals—protocol version 5.3.

[18] Higgins JP, Thompson SG, Deeks JJ, Altman DG (2003). Measuring inconsistency in meta-analyses. BMJ.

[19] Olivier C, Kunneke H, O’Connell N, Von Delft E, Wates M, Dramowski A (2018). Healthcare-associated infections in paediatric and neonatal wards: a point prevalence survey at four South African hospitals. S Afr Med J.

[20] Nair A, Steinberg WJ, Habib T, Saeed H, Raubenheimer JE (2018). Prevalence of healthcare-associated infection at a tertiary hospital in the Northern Cape Province, South Africa. S Afr Fam Pract.

[21] Mpinda-Joseph P, Anand Paramadhas BD, Reyes G, Maruatona MB, Chise M, Monokwane-Thupiso BB, Souda S, Tiroyakgosi C, Godman B (2019). Healthcare-associated infections including neonatal bloodstream infections in a leading tertiary hospital in Botswana. Hosp Pract.

[22] Abubakar U (2020). Point-prevalence survey of hospital acquired infections in three acute care hospitals in northern Nigeria. Antimicrob Resist Infect Control.

[23] Labi AK, Obeng-Nkrumah N, Owusu E, Bjerrum S, Bediako-Bowan A, Sunkwa-Mills G, Akufo C, Fenny AP, Opintan JA, Enweronu-Laryea C, Debrah S (2019). Multi-centre point-prevalence survey of hospital-acquired infections in Ghana. J Hosp Infect.

[24] Razine R, Azzouzi A, Barkat A, Khoudri I, Hassouni F, CharifChefchaouni A, Abouqal R (2012). Prevalence of hospital-acquired infections in the university medical center of Rabat, Morocco. Int Arch Med.

[25] Mahjoub M, Bouafia N, Bannour W, Masmoudi T, Bouriga R, Hellali R, Cheikh AB, Ezzi O, Abdeljellil AB, Mansour N (2015). Healthcare-associated infections in a Tunisian university hospital: from analysis to action. Pan Afr Med J.

[26] Ayed HB, Yaich S, Trigui M, Jemaa MB, Hmida MB, Karray R, Kassis M, Mejdoub Y, Feki H, Jedidi J, Damak J (2019). Prevalence and risk factors of health care-associated infections in a limited resources country: a cross-sectional study. Am J Infect Control.

[27] Jamoussi A, Ayed S, Ismail KB, Chtara K, Bouaziz M, Mokline A, Messaadi A, Merhebene T, Tilouche N, EL Atrous S, Boussarsar M (2018). The prevalence of healthcare-associated infection in medical intensive care units in Tunisia. Results of the multi-centre nosorea1 study. Tunis Med.

[28] Chiguer M, Alami Z, Lamti S, Abda N. Prevalence and risk factors of healthcare-associated infections in a Moroccan teaching hospital. Can J Infect Control. 2018;33(4).

[29] Ketata N, Ayed HB, Hmida MB, Trigui M, Jemaa MB, Yaich S, Maamri H, Baklouti M, Jedidi J, Kassis M, Feki H (2021). Point prevalence survey of health-care associated infections and their risk factors in the tertiary-care referral hospitals of Southern Tunisia. Infect Dis Health.

[30] Greco D, Magombe I (2011). Hospital acquired infections in a large north Ugandan hospital. J Prev Med Hyg.

[31] Yallew WW, Kumie A, Yehuala FM (2016). Point prevalence of hospital-acquired infections in two teaching hospitals of Amhara region in Ethiopia. Drug Healthc Patient Saf.

[32] Bunduki GK, Feasey N, Henrion MY, Noah P, Musaya J (2021). Healthcare-associated infections and antimicrobial use in surgical wards of a large urban central hospital in Blantyre, Malawi: a point prevalence survey. Infect Prev Pract.

[33] Alemu AY, Endalamaw A, Belay DM, Mekonen DK, Birhan BM, Bayih WA (2020). Healthcare-associated infection and its determinants in Ethiopia: a systematic review and meta-analysis. PLoS ONE.

[34] Alemu AY, Endalamaw A, Bayih WA (2020). The burden of healthcare-associated infection in Ethiopia: a systematic review and meta-analysis. Trop Med Health.

[35] World Health Organization (2015). Worldwide country situation analysis: response to antimicrobial resistance: summary.

[36] Rothe C, Schlaich C, Thompson S (2013). Healthcare-associated infections in sub-Saharan Africa. J Hosp Infect.

[37] Abubakar U, Syed Sulaiman SA, Adesiyun AG (2018). Utilization of surgical antibiotic prophylaxis for obstetrics and gynaecology surgeries in northern Nigeria. Int J Clin Pharm.

[38] Abubakar U, Sulaiman SA, Adesiyun AG (2020). Knowledge and perception regarding surgical antibiotic prophylaxis among physicians in the department of obstetrics and gynecology. Trop J Obstet Gynaecol.

[39] Abubakar U (2020). Antibiotic use among hospitalized patients in northern Nigeria: a multicenter point-prevalence survey. BMC Infect Dis.

[40] Abubakar U, Syed Sulaiman SA, Adesiyun AG (2019). Impact of pharmacist-led antibiotic stewardship interventions on compliance with surgical antibiotic prophylaxis in obstetric and gynecologic surgeries in Nigeria. PLoS ONE.

[41] Getahun H, Smith I, Trivedi K, Paulin S, Balkhy HH (2020). Tackling antimicrobial resistance in the COVID-19 pandemic. Bull World Health Organ.

[42] Rodríguez-Baño J, Rossolini GM, Schultsz C, Tacconelli E, Murthy S, Ohmagari N, Holmes A, Bachmann T, Goossens H, Canton R, Roberts AP (2021). Antimicrobial resistance research in a post-pandemic world: insights on antimicrobial resistance research in the COVID-19 pandemic. J Glob Antimicrob Resist.

[43] Ansari S, Hays JP, Kemp A, Okechukwu R, Murugaiyan J, Ekwanzala MD, Ruiz Alvarez MJ, Paul-Satyaseela M, Iwu CD, Balleste-Delpierre C, Septimus E (2021). The potential impact of the COVID-19 pandemic on global antimicrobial and biocide resistance: an AMR insights global perspective. JAC-Antimicrob Resist.

[44] Stevens MP, Doll M, Pryor R, Godbout E, Cooper K, Bearman G (2020). Impact of COVID-19 on traditional healthcare-associated infection prevention efforts. Infect Control Hosp Epidemiol.

[45] Knight GM, Glover RE, McQuaid CF, Olaru ID, Gallandat K, Leclerc QJ, Fuller NM, Willcocks SJ, Hasan R, van Kleef E, Chandler CI (2021). Antimicrobial resistance and COVID-19: intersections and implications. Elife.

[46] Abubakar U, Usman MN, Baba M, Sulaiman A, Kolo M, Adamu F, Jaber AA (2022). Practices and perception of healthcare workers towards infection control measures during the COVID-19 pandemic: a cross-sectional online survey from Nigeria. J Infect Dev Ctries.

[47] Muller LM, Gorter KJ, Hak E, Goudzwaard WL, Schellevis FG, Hoepelman AI, Rutten GE (2005). Increased risk of common infections in patients with type 1 and type 2 diabetes mellitus. Clin Infect Dis.

